# Accumulation of extra-chloroplastic triacylglycerols in *Arabidopsis* seedlings during heat acclimation

**DOI:** 10.1093/jxb/erv226

**Published:** 2015-05-14

**Authors:** Stephanie P. Mueller, Daniel M. Krause, Martin J. Mueller, Agnes Fekete

**Affiliations:** ^1^Julius-von-Sachs-Institute, Biocenter, Pharmaceutical Biology, University of Wuerzburg, D-97082 Wuerzburg, Germany

## Abstract

Heat acclimation enables plants to tolerate and survive short-term heat stress on hot days. In *Arabidopsis thaliana*, a genetically programmed heat shock response can be rapidly triggered in the temperature range of 32–38°C through activation of heat shock transcription factors (HSF). The heat shock response leads to heat acclimation and confers short-term protection against temperatures above 40°C. However, little is known about metabolic adjustments during heat acclimation.

Untargeted metabolite analyses of *A. thaliana* seedlings revealed that levels of polyunsaturated triacylglycerols (TG) rapidly and dramatically increase during heat acclimation. TG accumulation was found to be temperature dependent in a temperature range of 32–50°C (optimum at 42°C) and reversible after a return from 37°C to normal growth temperatures. Heat-induced TGs accumulated in extra-chloroplastic compartments and increased in both roots and shoots to a similar extent. Analysis of mutants deficient in all four *HSFA1* master regulator genes or the *HSFA2* gene revealed that TG accumulation was not dependent on HSFs. Moreover, the TG response was not limited to heat stress because drought and salt stress also triggered an accumulation of TGs, but not short-term osmotic, cold, and high light stress. Lipid analysis revealed that heat-induced accumulation of TGs was not due to massive *de novo* fatty acid synthesis. It is hypothesized that TGs serve as transient stores for fatty acids that may be required for membrane remodelling during heat acclimation.

## Highlight

Metabolite profiling of heat-acclimated seedlings revealed accumulation of raffinose and polyunsaturated triacylglycerols. Dynamic heat-induced triacylglycerol accumulation was independent of heat shock factors and appears to be involved in lipid remodelling.

## Introduction

Plants are exposed to daily and seasonal fluctuations of ambient temperatures. Growth at suboptimal temperatures requires short- and long-term adaptations of physiology and metabolism. During the day, temperatures are typically lowest in the morning and reach a maximum in the afternoon. Plants and other organisms have the inherent capacity to temporarily survive temperatures above the optimal growth temperature without prior acclimation (basal thermotolerance) and an ability to acquire tolerance to otherwise lethal heat temperatures (acquired thermotolerance) (Larkindale *et al.*, 2005). Basal thermotolerance may strongly vary depending on the plant species. For instance, the optimal growth temperature of *Arabidopsis thaliana* is 22°C but seedlings have been reported to tolerate higher temperatures for different time periods: 30°C up to 5 days (Chen *et al.*, 2006), 38°C for 16h (Clarke *et al.*, 2009) and 45°C for up to 45min (Yeh *et al.*, 2012). Short-term acquired thermotolerance (SAT) requires acclimation of *Arabidopsis* seedlings at moderately elevated temperatures of 32–38°C for 1h (Liu and Charng, 2012). During heat acclimation, a genetically programmed heat shock response (HSR) is triggered. This is characterized by rapid activation of heat shock transcription factors (HSF), which trigger a massive accumulation of a battery of heat shock proteins (HSP), chiefly involved in protein folding and protection. In addition, enzymes involved in redox regulation such as ascorbate peroxidase 2 (APX2) and metabolic enzymes such as galactinol synthase 1 (GOLS1) are strongly induced (Liu *et al.*, 2011). After acclimation at 38°C for 1.5h, seedlings survived exposure to 45°C for 2h, a condition that is lethal for non-acclimated plants (Hong and Vierling, 2000; Larkindale and Vierling, 2008). In nature, temperatures rise gradually during the day and, typically, there is sufficient time to acclimate in the temperature range 31–38°C before potentially lethal temperatures above 40°C are reached in the afternoon. Usually, high temperature stress lasts only a few hours before temperatures decrease during the night. SAT is a perfectly evolved mechanism that helps plants to cope with this situation. HSR genes are rapidly and transiently induced at moderately elevated temperatures and, hence, SAT is fully reversible and lasts for up to 72h (Charng *et al.*, 2006).

Plant HSFs comprise three evolutionary conserved classes: A, B, and C. The HSF class A possesses short peptide motifs enriched with aromatic and large hydrophobic amino acid residues embedded in acidic surrounding, which are essential for their activation (Kotak *et al.*, 2004). In *A. thaliana*, four *HSFA* genes belong to the *HSFA1* subclass (*HSFA1a*, *HSFA1b*, *HSFA1d*, and *HSFA1e*). HSFA1 are constitutively present and can be activated rapidly at moderately elevated temperatures. They have been termed the master regulators of the HSR because *hsfa1abd* triple and *hsfa1abde* quadruple mutants are unable to induce HSR or, therefore, acquire thermotolerance (Liu *et al.*, 2011; Yoshida *et al.*, 2011). Heat activation of *HSFA1* triggers the transcription of inducible HSFs. Among them, HSFA2 is required for the extension of short-term heat acclimation (Charng *et al.*, 2007) and mediates amplification of a subset of heat-responsive genes such as *HSP*s (including *HSP101, HSP26.5, HSP22-ER, HSP18.1, HSP70b*), *APX2*, and *GOLS1* as well as some drought- and cold-regulated genes (Schramm *et al.*, 2006).

Although regulation of metabolic enzymes after activation of HSR has been reported in several studies, limited information on heat-responsive metabolites could be found in the literature. After up-regulation of *GOLS1* at 37°C, raffinose and galactinol were found to accumulate rapidly in *Arabidopsis* leaves (Panikulangara *et al.*, 2004). As far as could be determined, only one large metabolite profiling analysis has been performed to study heat-induced changes of metabolic pathways in *Arabidopsis*. Studying basal thermotolerance at 40°C for up to 4h, Kaplan *et al.* (2004) identified 80 metabolite features that increased significantly *(P* < 0.05) after heat treatment for 2h. Only four known metabolites (galactinol, raffinose, pipecolic acid, and digalactosylglycerol) and four non-identified compounds showed a strong accumulation (fold change >4). However, short-term exposure to 40°C for only 1h can be lethal for *A. thaliana* seedlings (Larkindale and Knight, 2002) and, hence, the observed metabolic changes may reflect severe disturbance of cellular homoeostasis.

For identification of metabolites specifically regulated upon short-term moderate heat, the metabolome of 2-week-old *A. thaliana* seedlings grown at the optimal growth temperature (22°C) was compared with seedlings that were shifted to the optimal acclimation temperature (37°C) for 2h. To investigate the heat response on the metabolome level, untargeted metabolite analyses were performed using ultra-performance liquid chromatography (UPLC) coupled with a quadrupole time-of-flight mass spectrometer (qTOF-MS).

## Material and methods

### Plant material and growth conditions

*A*. *thaliana* wild-type ecotypes ‘Columbia-0’ and ‘Wassilewskija’ were grown in a growth chamber under an 8h/16h short-day cycle at 22°C (160 µE) for 2 weeks. Plants were grown on agar plates with Murashige & Skoog (MS) medium basal mixture including MES buffer (pH 5.7; Duchefa Biochemie BV, Haarlem, Netherlands), containing 3% sugar and 1.2% agarose. The mutant line *gols1* was kindly provided by Schoeffel (Panikulangara *et al.*, 2004); the *hsfa1abde* quadruple mutant was kindly provided by Ohama and Yamaguchi-Shinozaki (Yoshida *et al.*, 2011). Additionally, *hsfa2* (SALK_008978) was used.

### Heat treatment and abiotic stress treatments

For heat treatment, plants grown on agar plates (100 seeds per plate) were transferred 2h after the onset of light to a growth chamber set at 37°C for the time indicated. All seedlings grown on a plate were harvested, immediately shock frozen with liquid nitrogen, and stored at −80°C until extraction.

To determine the temperature dependency of the sugar and triacylglycerol (TG) markers, seedlings were transferred to liquid Murashige & Skoog media 1 day before the treatments. Eight seedlings were pooled and heated at different temperatures (22–50°C) in a heating block for 2h. For cold treatment, plates were transferred to a refrigerator set to 4°C for 2h. High light treatment was carried out by irradiating the seedlings with high quantum flux density (650 µE m^−2^ s^−1^) for 2h. For dehydration treatments, plants were gently removed from the plates and allowed to dry on paper for 2h. For osmotic and salt stress, seedlings were transferred to liquid Murashige & Skoog media 1 day before the experiment. Seedlings (eight seedlings were pooled) were treated with water, 125mM NaCl, or 250mM mannitol for 2h prior to harvesting.

### Chloroplast isolation

Chloroplasts in 3-week-old *A. thaliana* seedlings, grown on Murashige & Skoog agar containing 1% sucrose, were isolated according to Aronsson and Jarvis (2002). All manipulations were done at 4°C or on ice. Plants were transferred into 15mL of isolation buffer [20mM Hepes KOH, pH 8.0, 5mM ethylenediaminetetraacetic acid (EDTA), 5mM MgCl_2_ × H_2_O, 10mM NaHCO_3_, and 0.3M sorbitol], ground, and filtered three times in succession. The lysate was centrifuged at 950g for 4min and the pellet was gently resuspended in 2mL of isolation buffer. The sample was then layered on top of a discontinuous Percoll gradient (75% Percoll in gradient mix, 28% Percoll in gradient mix with 14% water; gradient mix: 25mM Hepes KOH, 10mM EDTA). After centrifugation at 1300g for 6min the green band that appeared at the interface of the Percoll layers was collected. The chloroplast-rich fraction was diluted with 10mL wash buffer (50mM HEPES KOH, pH 8.0, 3mM MgSO_4_, and 0.3M sorbitol) and centrifuged at 950g for 4min to remove the Percoll. Chloroplasts were resuspended in 200 µL wash buffer and a modified Bligh and Dyer extraction was carried out.

### Metabolite analysis

All solvents were at least HPLC grade and were purchased from Biosolve (Valkenswaard, Netherlands). Seedlings (100mg, pooled from one plate) were shock-frozen in liquid nitrogen and extracted two times with 600 µl of chloroform/methanol/water (3:2:1, v/v) using a ball mill at 21 Hz for 10min (Retsch, Haan, Germany). As internal standards (IS), 0.2 µg of α, α-[1,1’-D2] trehalose (Sigma Aldrich, Taufkirchen, Germany) and 0.24 µg of tridecanoyl-triacylglycerol (TG30:0; Larodan, Solna, Sweden) were added into a sample. The aqueous phase was used for the analysis of hydrophilic metabolites and the organic phase was used for lipid analysis. The organic phase was evaporated in a vacuum concentrator at 40°C. The residue was resuspended in 100 µL isopropanol for analysis.

For untargeted metabolite analysis, the organic and aqueous phases were analysed with an ACQUITY UPLC system coupled to a Synapt G2 HDMS qTOF-MS (all Waters, Eschborn, Germany). Chromatographic separation of the organic phase was carried out on a BEH C18 column (2.1×100mm, 1.7 μm; Waters) with a linear binary solvent gradient of 30−100% eluent B over 10min at a flow rate of 0.3mL min^−1^. Eluent A consisted of 10mM ammonium acetate in water/acetonitrile (60:40, v/v) and eluent B consisted of 10mM ammonium acetate in isopropanol/acetonitrile (90:10, v/v). Chromatographic separation of the aqueous phase was performed on a BEH amide column (1.7 µm, 2.1×100mm; Waters) according to the Waters application note WA60126 with modifications. Briefly, elution was performed using a linear solvent-strength gradient (0.2mL min^−1^ at 35°C) from 75% to 45% acetonitrile containing 1% ammonium hydroxide in 10min.

After chromatographic separation, hydrophilic and lipophilic metabolites were detected by MS coupled with an electrospray ionisation (ESI) source operated in positive and negative modes. The ESI capillary voltage was set to 0.8kV and nitrogen (at 350°C, flow rate of 800L h^−1^) was used as desolvation gas. The quadrupole was operated in a wide-band RF mode, and data was acquired over the mass range of 50−1200Da. Two discrete and independent interleaved acquisition functions were automatically created. The first function collected the low energy data where molecule ions were acquired while the second function collected the fragments of the molecule ion (high energy data) by using a collision energy ramp from 15 to 35eV (MS^E^). MassLynx, MarkerLynx, and QuanLynx (version 4.1; all Waters) were used to acquire and process chromatograms.

Response factors for TG54:9, TG54:6, TG54:3, TG54:0, TG51:0, and TG48:0 (Larodan) were determined using TG30:0 as IS. Because the response factors were in the range of 0.8–1.1, a response factor of 1 was used for semi-quantitative analysis of TGs. The inter-day repeatability of peak areas were <6% at a concentration of 100ng/sample (n = 3) and the determined linear range spanned two orders of magnitude (regression coefficient >0.995 between 10 and 1000ng/sample) using standard solutions of TG54:9, TG54:6, TG54:3, TG54:0, TG51:0, and TG48:0.

For the targeted analysis of the defined lipid species (Supplementary Table S1), glycerophosphoethanolamine (PE) 34:0, glycerophosphocholin (PC) 34:0, monogalactosyldiacylglycerol (MGDG) 36:0, digalactosyldiacylglycerol (DGDG) 36:0, and diacylglycerol (DG) 34:0 (2.4 µg/sample) were used as IS for each lipid class. For semi-quantitative analysis, peak areas of the analytes and ISs were determined in the extracted total ion chromatogram and lipid concentrations were calculated by using a response factor of 1 for each analyte/IS pair.

For the determination of raffinose and galactinol in the aqueous phases, multiple reaction monitoring (MRM) was applied using a Waters Micromass Quattro Premier triple quadrupole MS coupled to UPLC (Waters). The operational parameter of chromatographic separation was identical to the ones used for the untargeted metabolite analysis. The sugars were ionized in an ESI source operated in the negative mode. The collision-induced dissociation of each compound for MRM was performed using argon as the collision gas with a flow rate of 0.3mL min^−1^ and a pressure of 3.0×10^−3^ mbar. The following MRM transitions were monitored: m/z 503→179 at a retention time (RT) of 5.33min for raffinose, m/z 341→179 at RT of 5.79min for galactinol, and m/z 343→180 at RT of 4.51min for D2-trehalose (IS).

Chlorophyll content was measured according to Aronsson and Jarvis (2002). Briefly, the lipophilic extract was used and diluted 1 to 10 with isopropanol and measured at absorption wavelengths of 647nm and 664nm with NanoDrop1000 (Thermo Fisher Scientific, Dreieich, Germany).

For total fatty acid analysis, shock-frozen seedlings (100mg) were extracted with 500 µL of isopropanol containing 10% potassium hydroxide and 5 µg heptadecanoic acid (IS) in a ball mill (21 Hz, 10min). The supernatant was incubated at 60°C for 1h. After centrifugation, the pH of the supernatant was adjusted to 6 and analysed by UPLC–qTOF-MS (Waters) in the negative ESI mode. Analytes were eluted on a BEH C18 column (1.7 µm, 2.1×100mm; Waters) at 40°C using an eluent from 70% to 100% acetonitrile (acidified with 0.1% formic acid) within 10min at a flow rate of 0.3mL min^−1^. The fatty acid RTs, mass-to-charge ratios (m/z), and response factors are included in Supplementary Table S1.

## Results

### Untargeted metabolite analysis revealed accumulation of raffinose, galactinol, and polyunsaturated triacylglycerols in heat-acclimated seedlings

**Fig. 1.**
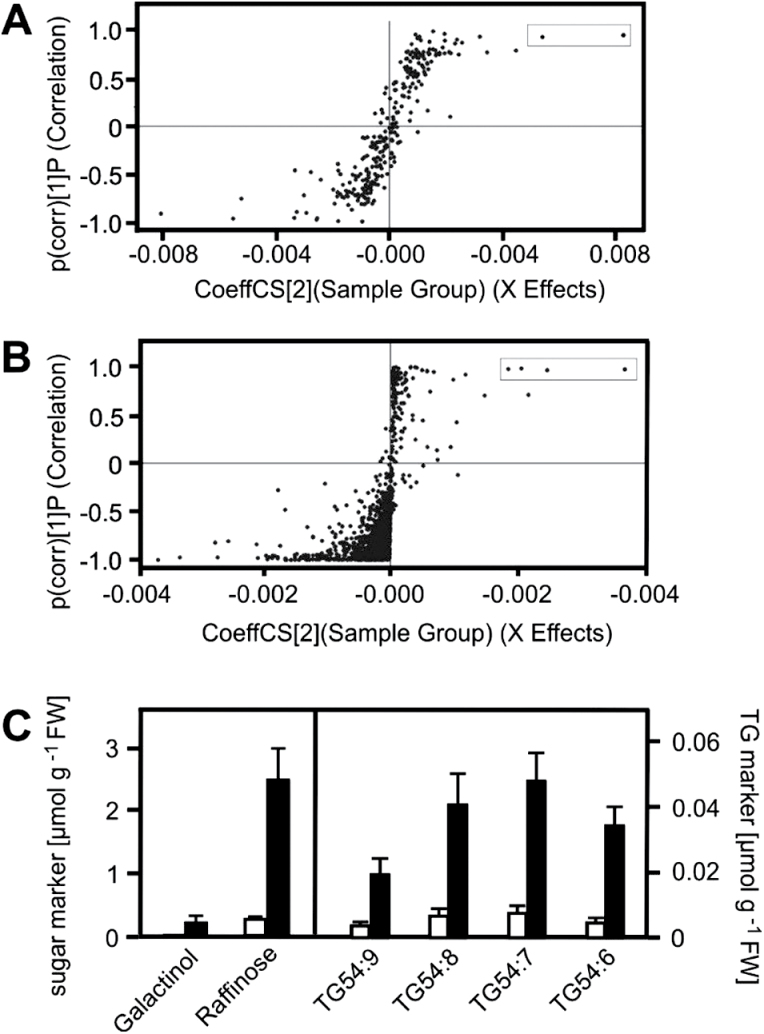
Identification of heat-responsive hydrophilic and lipophilic metabolites using orthogonal partial least square discriminant analysis (OPLS-DA). Two-week-old control (22°C) and heat-acclimated (37°C, 2h) seedlings grown on agar plates were extracted with liquid-liquid extraction. OPLS-DA was performed on the detected hydrophilic (A) and lipophilic (B) metabolite features. Significantly heat-induced metabolites are presented in boxes and were identified as raffinose, galactinol, and polyunsaturated TG species (C). Levels of the sugar and TG markers in control (white columns) and heat-acclimated (black columns) seedlings are shown (C). Data represent means ± SD, n = 9.

In order to identify metabolic pathways responding to elevated temperature, global metabolite profiles of heat-acclimated and control seedlings were compared. Two-week-old *A. thaliana* seedlings grown on agar plates were shifted to 37^o^C for 2h (Supplementary Fig. S1). Metabolites of heat-treated and untreated seedlings were isolated with liquid-liquid extraction to recover hydrophilic and lipophilic metabolites simultaneously. Hydrophilic metabolites recovered in the aqueous phase were analysed by hydrophilic interaction liquid chromatography coupled to MS and lipophilic metabolites in the organic phase were separated by reversed phase liquid chromatography coupled to MS. In total, 248 and 987 metabolite features were detected in the aqueous and organic phases, respectively. Discrimination in the metabolome of treated and control seedlings was observed using principal component analysis (Supplementary Fig. S2). Orthogonal partial least square discriminant analysis revealed significant accumulation of two hydrophilic and four lipophilic metabolites (termed as markers) in the acclimated seedlings (Fig. 1).

The structures of the six identified markers were identified by comparing their accurate masses and fragmentation patterns with metabolome databases (Supplementary Table S2). The Metabolite and Tandem MS Database (METLIN) listed 45 and 34 hits for the two hydrophilic metabolites displaying m/z of 503.161 and 341.108, respectively. After determining the elemental compositions of the two heat-responsive hydrophilic metabolites by using isotope abundance distribution, all hits were identified as oligosaccharide derivatives. Literature on plant heat-stress responses suggested raffinose and galactinol as possible metabolites, which was confirmed by comparing the RTs and fragmentation patterns of the markers to authentic reference materials.

The four lipophilic markers with m/z of 890.7238, 892.7394, 894.7551, and 896.7707 were identified as ammonium adducts of TG using METLIN and the Database of Lipid Metabolites and Pathways Strategy (LIPID MAPS). In addition, collision-induced fragmentation of the lipophilic markers revealed a loss of fatty acyls, resulting in abundant formation of diacylglycerol (DG) ions and protonated fatty acid fragments (Supplementary Table S2) that are characteristic for TGs (Murphy *et al.*, 2007). The four heat-responsive TGs were identified (Supplementary Table S2) as trioctadecatrienoyl-glycerol (TG54:9), dioctadecatrienoyl-octadecadienoyl-glycerol (TG54:8), dioctadecadienoyl-octadecatrienoyl-glycerol (TG54:7), and octadecenoyl-octadecadienoyl-octadecatrienoyl-glycerol (TG54:6). The structure elucidation was confirmed using commercially available TG54:9 and TG54:6 as reference compounds.

### Accumulation of predominantly polyunsaturated TG species after heat acclimation

**Fig. 2.**
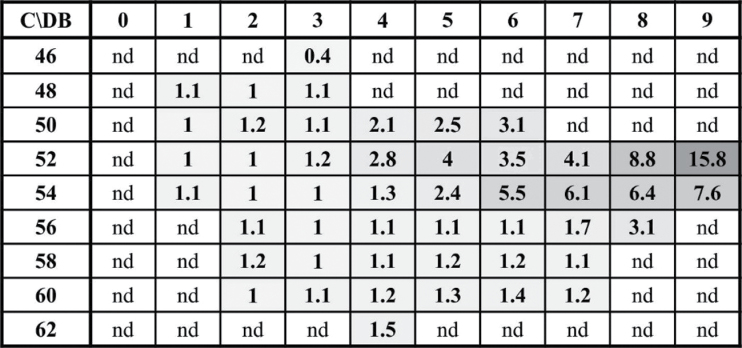
Fold increase of identified TGs after heat acclimation. TGs were plotted according to the number of carbons (rows) and double bonds (columns) of the fatty acids esterified to glycerol. Fold changes after heat acclimation (37°C, 2h) are shown. C = number of carbon atoms in the fatty acyls, DB = number of double bonds in the fatty acyls, nd = non-detected. Data represent means ± SD, n=9.

To investigate if TG species other than the TG markers accumulated during heat acclimation (37°C, 2h), endogenous TGs were identified and quantified using an in-house developed database (unpublished). RT-aligned molecule ion and fragment spectra led to the identification of 48 TGs specified by the number of carbons and double bonds of the acyl chains. Determination of (Supplementary Table S3) all RT-aligned fragments resulting from neutral losses of fatty acyls in the pre-processed data were obligatory. After short validation of the analysis method, levels of the 48 TGs were determined in heat-acclimated and control seedlings (Supplementary Table S3). Out of the 48 TG species, 17 increased by 1.5- to 16-fold at 37°C (Fig. 2). Out of the most abundant TG species (levels higher than 2.5 mol%), the four most strongly induced species were the TG markers (TG54:9, TG54:8, TG54:7, and TG54:6). By contrast, no significant accumulation was observed for TG species comprising saturated and/or very long chain fatty acyls. Thus, to characterize the TG response to heat acclimation, we determined total levels of TG markers in the following experiments.

### Time- and temperature-dependent accumulation of sugar and TG markers in response to elevated temperature

**Fig. 3.**
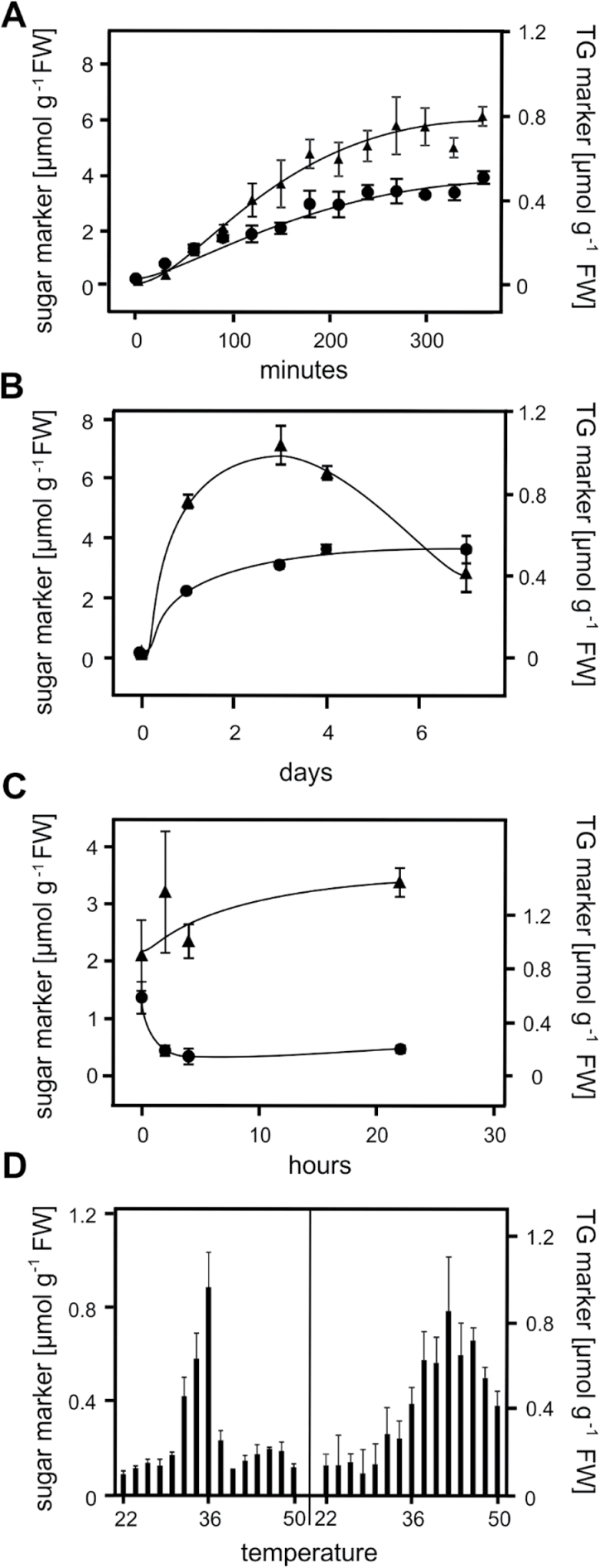
Time- and temperature-dependent responses of sugar (▲) and TG (●) markers in *Arabidopsis* seedlings. Seedlings exposed to 37°C for the times indicated were analysed (A and B). Heat-acclimated seedlings (37°C, 2h) were transferred back to the growth condition (22°C) for the times indicated (C). Sugar and TG markers were determined in seedlings treated at different temperatures (22°C to 50°C) for 2h (D). Data represent means ± SD, n = 4.

To study the time dependency of the marker responses, sugar and TG markers were determined in 2-week-old *Arabidopsis* seedlings after treatment at 37°C for different exposure times (Fig. 3A). Already after 30min, a 6-fold increase in the levels of the sugar and TG markers was determined. Levels of the markers steadily increased up to 6h. Upon long-term exposure to 37°C (Fig. 3B), levels of TG markers remained high for at least 7 days while levels of the sugar markers declined after 4 days. When heat-acclimated seedlings (37°C, 2h) were returned to 22°C (Fig. 3C), levels of sugar markers remained constant for at least 24h while levels of TG markers dropped to nearly basal levels after 2h.

To investigate if the responses of the markers were dependent on the temperature, seedlings in liquid media were treated at different temperatures ranging from 22°C to 50°C for 2h (Fig. 3D). All markers accumulated at a temperature above 30°C. The highest accumulation was determined at 36°C for the sugar markers (0.8 µmol g^−1^ fresh weight) and 42°C for the TG markers (0.85 µmol g^−1^ fresh weight). Interestingly, the sugar markers did not accumulate at temperatures above 38°C. By contrast, an increase of the TG markers was observed even at 50°C.

### Significant accumulation of TGs after short-term heat, drought, and salt stress but not after short-term cold, osmotic, or high light stress

**Fig. 4.**
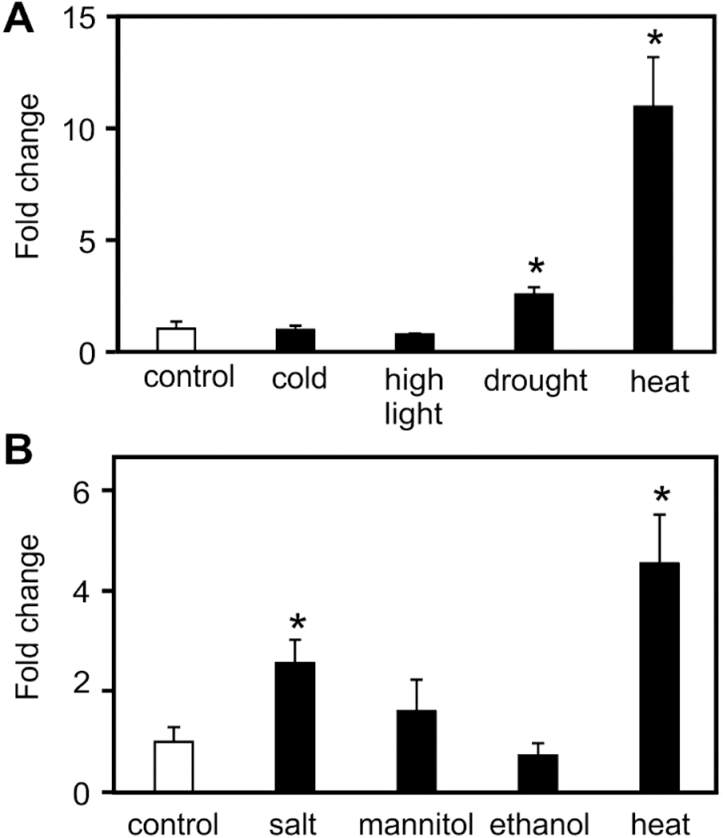
Accumulation of TG markers after different abiotic stresses. Two-week-old seedlings grown on agar plates were exposed to cold (4°C, 2h), high light (900 µE m^−2^ s^−1^, 2h), drought (seedlings placed on filter paper), or heat (37°C, 2h) stress (A). Seedlings grown in liquid Murashige & Skoog medium were treated with salt (125mM NaCl, 2h), mannitol (250mM, 2h), ethanol (1%, 2h), or heat (37°C, 2h) stress (B). Data represent means ± SD, n = 4.

Raffinose has been reported to increase after several abiotic stresses such as heat, cold, drought, salt, and oxidative stress (Taji *et al.*, 2002; Nishizawa *et al.*, 2008). Therefore, accumulation of TG markers was examined to determine if it is a general stress response or specifically up-regulated by heat. Two-week-old seedlings grown on agar plates were subjected to low temperature (4°C) as well as elevated light intensity (900 µE m^−2^ s^−1^) and drought (seedlings were placed on filter paper) for 2h (Fig. 4A). In another series of experiments, seedlings were transferred to liquid Murashige & Skoog media and exposed to salt stress (125mM NaCl), osmotic stress (250mM mannitol), and 1% ethanol for 2h (Fig. 4B). As shown in Fig. 4, heat appeared to be the strongest inducer of TG markers under these test conditions. However, levels of TG markers were also significantly increased after salt and drought stress (2.6- and 2.5-fold, respectively). By contrast, no significant induction of TG markers was determined after osmotic, cold, high light, or ethanol treatment.

### Heat-induced TG accumulation is independent of HSFs and raffinose biosynthesis

**Fig. 5.**
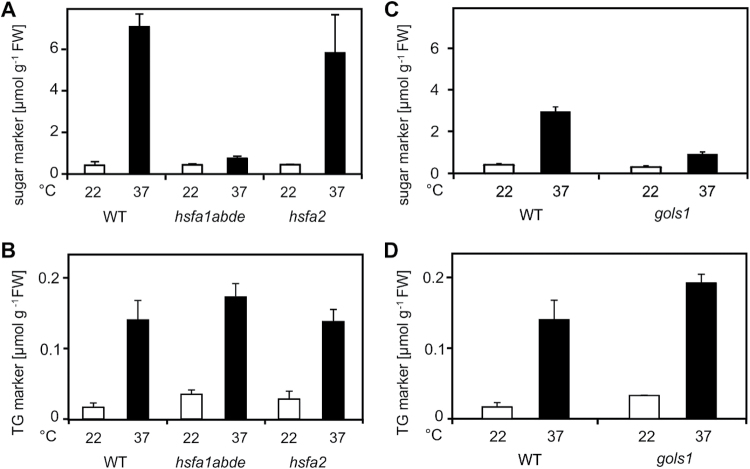
Accumulation of TG markers in *hsfa1abde*, *hsfa2*, and *gols1* mutant *Arabidopsis* seedlings after heat acclimation. Levels of sugar and TG markers were determined in 2-week-old *hsfa1abde*, *hsfa2*, and *gols1* seedlings kept at 22°C (white bar) and 37°C for 2h (black bar). Data represent means ± SD, n = 4.

In order to determine whether the accumulation of sugar and TG markers after heat acclimation is induced in an HSF-dependent fashion, mutant *Arabidopsis* plants lacking HSFA1 or HSFA2 were examined. No significant accumulation of raffinose was determined in the *hsfa1abde* quadruple mutant, while raffinose levels increased to wild-type levels in the *hsfa2* mutant when seedlings were treated at 37°C for 2h (Fig. 5A). By contrast, accumulation of TG markers was not dependent on HSFA1 and HSFA2 transcription factors (Fig. 5B). To investigate if the accumulation of TG markers is dependent on raffinose biosynthesis, the *gols1* mutant deficient in heat-induced raffinose synthesis was characterized (Fig. 5C). Levels of the TG markers in heat-acclimated *gols1* were similar to levels in the heat-acclimated wild type (Fig. 5D). These results indicate that TG markers accumulate independently of HSFs and of raffinose biosynthesis.

### TG markers accumulate predominantly in extra-chloroplastic compartments

**Fig. 6.**
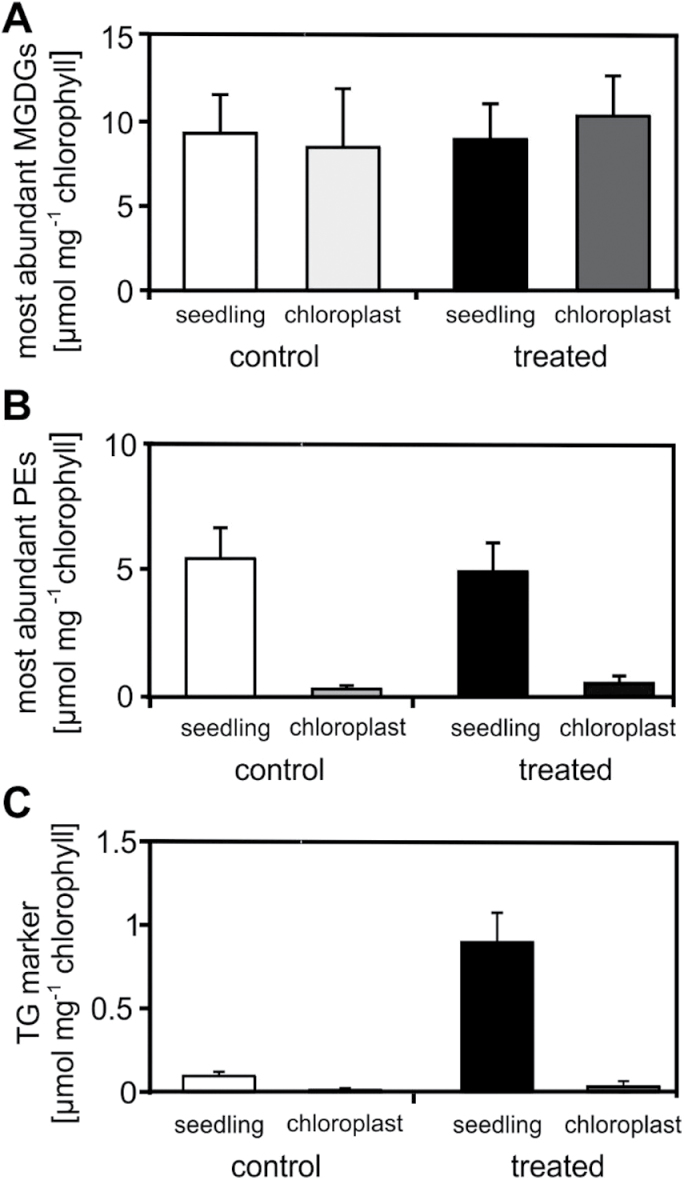
TG markers accumulated predominantly in extra-chloroplastic compartments after heat acclimation. Chloroplasts of control (white columns) and heat-acclimated (37°C, 2h; black columns) seedlings were isolated and most abundant MGDGs (A), PEs (B), and TG markers (C) were determined relative to chlorophyll levels. The determined MGDGs and PEs were MGDG36:6, MGDG34:4, PE36:5, PE36:4, PE34:3, and PE34:2. Data represent means ± SD, n = 3.

**Fig. 7.**
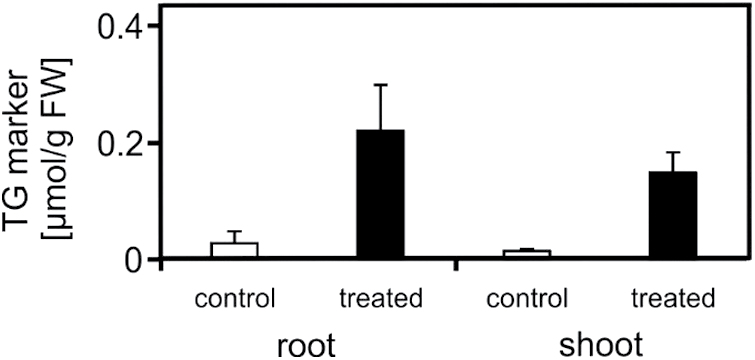
Levels of TG markers in roots and shoots. Roots and shoots of control (22°C, white columns) and heat-acclimated (37°C, 2h, black columns) seedlings were harvested; shoots were separated from roots and TG markers were analysed. Data represent means ± SD, n = 4.

In *Arabidopsis* leaves, TGs may either accumulate in the cytosol or chloroplasts. To localize the subcellular accumulation of TGs after heat acclimation, TG markers were determined in isolated chloroplasts and whole seedlings. Levels of TG markers as well as compartment-specific lipids were determined and normalized to chlorophyll content to compare the two different preparations. The reliability of the chloroplast isolation was confirmed by analysing the most abundant plastid-specific monogalactosyldiacylglycerols (MGDGs) with the acyl combinations 18:3–18:3 and 18:3–16:3. As expected, no significant differences in the levels of the two most abundant MGDG species between seedlings and chloroplast fractions were determined (Fig. 6A). To further assess the purity of the preparations, the most abundant PEs (PE36:5, PE36:4, PE34:3, PE34:2), which do not occur in plastids, were analysed. The contamination of PEs in the chloroplast fractions was <5% (Fig. 6B). TG markers accumulated 3.5-fold in the chloroplasts and 9-fold in the extra-chloroplastic compartments (Fig. 6C). TG markers were determined at a concentration of 32 nmol mg^−1^ chlorophyll in chloroplasts and 900 nmol mg^−1^ chlorophyll in seedlings after heat treatment (Supplementry Table S4). Considering the contamination of the chloroplast fraction with extra-plastidic components (5%), results suggest that the vast majority of TG markers accumulated in the cytosol. In addition, TG markers were analysed separately in shoots and roots. Interestingly, there was a similar increase in levels (8- to 10-fold) and similar absolute levels in both tissues after heat acclimation (Fig. 7).

### Total membrane lipid composition remained unaltered after heat acclimation

**Fig. 8.**
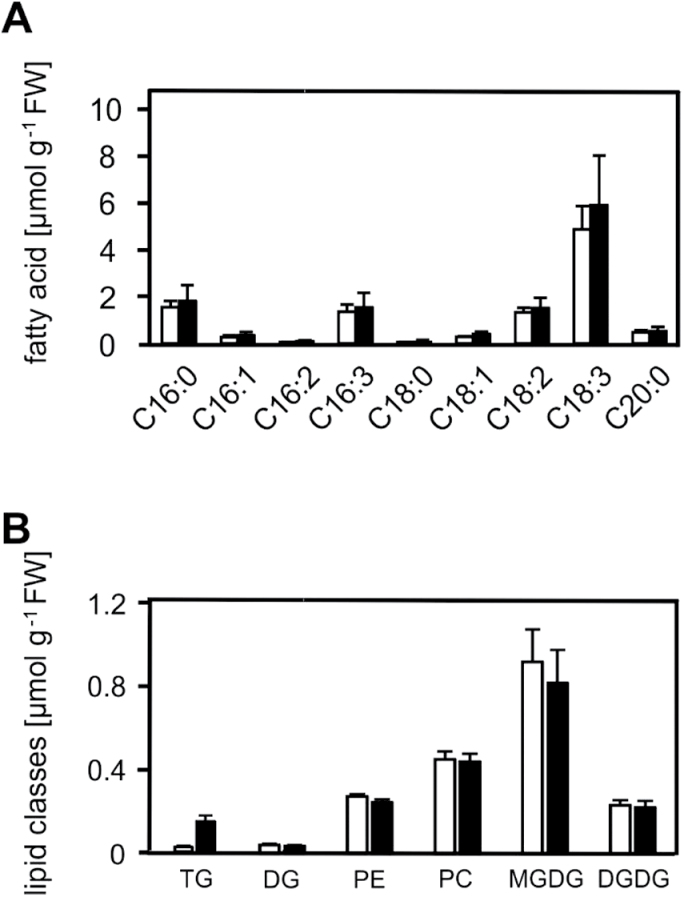
Levels of total fatty acids, neutral lipids, and membrane lipids after heat acclimation. Total fatty acids (A), TG markers and complex lipids (B) from control (22°C; white columns) and heat-acclimated (37°C, 2h; black columns) seedlings. Data represent means ± SD, n = 4 for the determination of total fatty acids, and n = 9 for the determination of complex lipids.

Rapid heat-induced TG accumulation could be either due to increased TG biosynthesis or reduced TG breakdown. With respect to TG biosynthesis, TGs can be synthesized by *de novo* synthesis of fatty acids and incorporation into TGs through the Kennedy pathway, or by channelling fatty acids from structural lipids to TGs (membrane remodelling). To this end, total fatty acids in heat-acclimated (37°C, 2h) and control seedlings (22°C) were determined. Levels of total fatty acids (esterified and free fatty acids) in heat-acclimated and control seedlings were comparable (Fig. 8A). To test whether fatty acids required for TG synthesis are derived from membrane lipid breakdown, levels of membrane lipids including MGDGs, DGDG, PEs, and PC as well as DG and TGs were determined (Fig. 8B and Supplementary Table S5). Except for TG markers, however, no significant change in the lipid composition was observed after heat acclimation.

## Discussion

### Rapid TG accumulation during heat acclimation

Untargeted and targeted metabolite analyses were performed to identify metabolic pathways that were regulated during heat acclimation. There was a dramatic accumulation of the sugars raffinose and galactinol as well as of the most abundant TG species within 30min after a temperature shift from 22°C to 37°C. Accumulation of raffinose and galactinol after moderate heat has already been described (Kaplan *et al.*, 2004; Panikulangara *et al.*, 2004). In addition, Kaplan *et al.* identified pipecolic acid and digalactosylglycerol as heat-induced metabolites in 5-week-old *Arabidopsis* plants at 40°C (Kaplan *et al.*, 2004). These metabolites, however, were not increased in this study using 2-week-old *A. thaliana* seedlings after 2h at 37°C. As far as can be determined, the massive accumulation of TGs after heat acclimation in *A. thaliana* has not been characterized before. Levels of TG markers increase extremely rapidly and were sensitive to temperature increases (Fig. 3D). Moreover, levels of TG markers rapidly dropped after the return to basal temperatures (Fig. 3C). Notably, the heating method (growth chamber or heating block) and growth conditions (seedlings grown on agar plates or transferred in liquid medium 1 day before treatment) as well as the time to harvest the seedling outside the growth chamber had a strong impact on TG levels. Therefore, some variations of mean TG levels in different experiments may reflect differences in experimental conditions.

The raffinose pathway is known to be up-regulated by a variety of stresses in the absence of heat. For instance, drought, salt, and cold stress are induced by expression of *GOLS1* or *GOLS3*, leading to the accumulation of raffinose and galactinol (Taji *et al.*, 2002). While TG accumulation was triggered by drought and salt stress, other short-term stresses such as cold, high light, and osmotic stress did not induce a significant accumulation of TGs in *Arabidopsis* seedlings (Fig. 4). It has been shown that TGs increase upon long-term cold treatment (Moellering *et al.*, 2010), desiccation (Gasulla *et al.*, 2013), senescence (Watanabe *et al.*, 2013), and *Pseudomonas* infection (Zoeller *et al.*, 2012) in *Arabidopsis*. Collectively, data suggest that accumulation of TGs, similar to the raffinose response, is a stress response that is not limited to elevated temperatures.

### Heat-induced TG accumulation is not part of the genetically programmed HSR

The four master HSFA1s are essential for up-regulation of a series of heat-inducible HSFs and HSPs as well as anti-oxidative enzymes such as APX2 and metabolic enzymes such as GOLS1 (Liu *et al.*, 2011). Out of the four HSFA1s, only HSFA1a and b are essential for rapid heat-induced transcription of *GOLS1* and raffinose accumulation, because *GOLS1* expression levels are strongly reduced in *hsf1ab* double mutants (Busch *et al.*, 2005). In agreement with these findings, a lack of raffinose accumulation in heat-treated *hsfa1abde* was observed (Fig. 5A). In addition, HSFA2, a heat- and stress-induced transcription factor, also contributes to *GOLS1* expression because it is reduced in heat-treated *hsfa2* mutant plants (Busch *et al.*, 2005; Nishizawa *et al.*, 2006). Transgenic plants overexpressing *HSFA2* overexpress *GOLS1* and over-accumulate raffinose and galactinol (Nishizawa *et al.*, 2008). However, under the experimental conditions in this study, *hsfa2* mutant seedlings still accumulated wild-type levels of raffinose during heat acclimation (Fig. 5A). *Hsfa1* and *hsfa2* mutant plants displayed wild-type-like accumulation of TG markers in response to heat (Fig. 5B), suggesting that TG accumulation is not part of the genetically programmed HSR. Additionally, *gols1* mutant plants that are unable to accumulate raffinose and galactinol did not over-accumulate TGs in response to heat (Fig. 5D), suggesting that carbons are not re-directed from the sugar pathway to the TG pathway.

### TG accumulation occurs in the cytosol of *Arabidopsis* seedlings

TG biosynthesis and accumulation has been best studied in *Arabidopsis* seeds but also occurs in mature leaves. In leaves, TGs have been identified in thylakoid-associated plastoglobuli in chloroplasts as well as in oil bodies in the cytosol (Martin and Wilson, 1984; Lippold *et al.*, 2012). Plastoglobuli are lipid bodies, containing mainly TGs, carotenoids, and prenyl quinones (Steinmuller and Tevini, 1985). Their swelling and formation is often associated with stress responses such as drought (Eymery and Rey, 1999), nitrogen starvation (Gaude *et al.*, 2007), and, notably, moderate heat stress (Zhang *et al.*, 2010). In plastids, phytyl ester synthases 1 and 2 can acylate DGs and might be involved in TG synthesis in chloroplasts (Lippold *et al.*, 2012). Given that plastoglobuli have been shown to accumulate upon abiotic stress responses, in particular after heat stress, heat-responsive TGs may accumulate in chloroplasts. However, lipid analyses of isolated chloroplasts and seedlings revealed that TG markers did not accumulate in chloroplasts, suggesting cytosolic assembly and storage of TGs after heat stress (Fig. 6). In addition, TGs accumulate to a similar extent in roots and shoots after heat acclimation (Fig. 7), indicating extra-chloroplastic storage of TGs. Similar to TGs, raffinose is also synthesized in the cytosol (Schneider and Keller, 2009) and is also heat inducible in roots (Panikulangara *et al.*, 2004).

Hence, TGs are likely synthesized through cytosolic pathways. Enzymes of the endoplasmic reticulum–localized Kennedy pathway involved in TG biosynthesis, like acyl-CoA:G3P acyltransferase, lysophosphatidic acid acyltransferase, and DG acyltransferase catalyse the sequential acylation of glycerol in developing seeds. An alternative TG biosynthesis pathway in the endoplasmic reticulum involves acyl-CoA independent transacylase phospholipid: DG acyltransferase that converts PCs and DGs to TGs (Chapman and Ohlrogge, 2012).

In order to clarify if accumulation of TG markers originates from *de novo* synthesis, levels of free and esterified fatty acids were analysed. Levels of total fatty acids (free and esterified) did not significantly change during heat acclimation (Fig. 8A), suggesting that TG accumulation is not driven by massive *de novo* fatty acid synthesis. Alternatively, accumulation of TG markers may reflect lipid remodelling, i.e. channelling of fatty acids from constitutive membrane lipids to TGs. However, significant changes were not observed in most abundant membrane lipid species, including PCs, MGDGs, DGDGs, PEs, and DGs after heat acclimation (Fig. 8B). These analyses indicate that a dramatic disturbance of membrane lipid homeostasis did not occur. However, the fatty acid pool in TGs was 1–8% compared to the total fatty acid pool in structural membrane lipids and, hence, fine-tuning of lipid pools might have escaped detection. Because TG markers predominantly contain polyunsaturated fatty acids, it is likely that polyunsaturated fatty acids are released from structural lipids and used for transient assembly of TGs during membrane remodelling.

After short-term heat treatment, slightly altered levels of MGDGs have been reported that did not result in discrimination when using multivariate statistical analysis (Burgos *et al.*, 2011). Therefore, small but potentially relevant changes in absolute lipid levels might have escaped detection. Another possibility is that heat-induced accumulation of TGs simply reflects decreased degradation and turnover of the cellular TG pool due to reduced fatty acid consumption during heat stress. It remains to be clarified how the TG pool is regulated through transcriptional or post-transcriptional mechanisms. Notably, transcripts of key proteins involved in cytosolic TG biosynthesis, such as phospholipid: DG acyltransferase 1 and DG acyltransferase, are not up-regulated during heat acclimation (Hruz *et al.*, 2008), indicating that rapid modulation of the TG pool may be regulated by post-transcriptional events.

### Functional significance of heat-induced raffinose and TG accumulation

Raffinose has been proposed to act as an osmoprotective solute (Panikulangara *et al.*, 2004) and an antioxidant (Nishizawa *et al.*, 2008) in abiotic stresses. *Arabidopsis* transgenic plants that constitutively overexpress *GOLS1* or *GOLS2* and accumulate raffinose and galactinol are more resistant to cold, salt, oxidative, and drought stress (Taji *et al.*, 2002; Nishizawa *et al.*, 2008). However, *gols1*-mutant plants that are unable to accumulate raffinose and galactinol after heat stress were fully competent to acclimate to heat stress (Panikulangara *et al.*, 2004). Hence, up-regulation of the raffinose pathway appears to protect plants from a variety of abiotic stresses but not from heat stress.

The biological significance of TG accumulation during heat stress is not known. Previous studies have demonstrated activation of TG synthesis and a concurrent decrease in galactolipids and phospholipids in ozone-fumigated spinach leaves (Sakaki *et al.*, 1990*a*, *b*; Sakaki *et al.*, 1990c), drought-stressed cotton leaves (El-Hafid *et al.*, 1989), and cold-stressed (Moellering *et al.*, 2010) as well as senescent *Arabidopsis* plants (Kaup *et al.*, 2002; Watanabe *et al.*, 2013). Severe stresses may trigger degradation of structural membrane lipids with small head groups like MGDGs and PCs through induction of galactolipases and phospholipases (Kaup *et al.*, 2002; Welti *et al.*, 2002; Moellering *et al.*, 2010). The degradation products like free fatty acids and DGs may then be converted to TGs. An accumulation of DGs, which form inverted micellar structures, may introduce small areas with unstable negative curvatures into bilayers that can lead to the fusion of apposed membrane bilayers (Goni and Alonso, 1999; Gasulla *et al.*, 2013). In addition, high levels of free fatty acids that display detergent-like amphiphilic properties are not well tolerated by plant cells. Thus, removal of DGs and fatty acids by formation of TG-rich oil bodies might contribute to membrane stabilization (Moellering *et al.*, 2010; Gasulla *et al.*, 2013). It has also been proposed that fatty acids derived from thylakoid galactolipid breakdown are transiently stored in TGs prior to conversion into phloem-mobile sucrose (Kaup *et al.*, 2002; Watanabe *et al.*, 2013). To clarify a possible function of TG accumulation for heat acclimation and resistance, stress resistance of transgenic plants that over-accumulate TGs or are deficient in stress-induced TG accumulation remains to be investigated.

## Supplementary data

Supplementary data are available at *JXB* online.

Fig. S1. Thermotolerance assay. Schemes of heat stress regimes for the thermotolerance assay is shown in panel A. *Arabidopsis* seedlings (100 seeds/plate) were grown at 22°C (a) and heat acclimated at 37°C for 2h (b). Acclimated (c) and non-acclimated (d) seedlings were then shifted to 45°C for 2h. The survival rate was measured 5 days after the heat treatment (B). Data represent means ± SD, n = 4.

Fig. S2. Principal components analysis (PCA) revealed a discrimination in the metabolome of heat-acclimated and control seedlings. Metabolite features of the hydrophilic (A) and hydrophobic (B) compounds were subjected to PCA. The first component in the PCA plots differentiated the metabolite features of heat-acclimated from control seedlings (n = 4).

Table S1. Identification parameters (m/z and RT) of identified lipids.

Table S2. Parameters used for the identification of heat-responsive markers.

Table S3. Amount of the identified TGs in control and heat acclimated (37°C, 2h) seedlings. Data represent means ± SD, n = 9.

Table S4. Lipid analysis of control and heat acclimated (37°C, 2h) samples in chloroplast fractions and in seedlings. Levels of the most abundant MGDGs (A), PEs (B), and TG markers (C) were determined in total seedling extracts and isolated chloroplasts and normalized to chlorophyll content. Data represent means ± SD, n = 3.

Table S5. Levels of complex lipids in heat-acclimated (37°C, 2h) and control seedlings (22°C). Data represent means ± SD, n = 9.

Supplementary Data

## References

[CIT0001] Aronsson H Jarvis P 2002 A simple method for isolating import-competent *Arabidopsis* chloroplasts. FEBS Letters 529, 215–220. 12372603 10.1016/s0014-5793(02)03342-2

[CIT0002] Burgos A Szymanski J Seiwert B Degenkolbe T Hannah MA 2011 Analysis of short-term changes in the *Arabidopsis thaliana* glycerolipidome in response to temperature and light. The Plant Journal 66, 656–668. 21309866 10.1111/j.1365-313X.2011.04531.x

[CIT0003] Busch W Wunderlich M Schoffl F 2005 Identification of novel heat shock factor-dependent genes and biochemical pathways in *Arabidopsis thaliana* . The Plant Journal 41, 1–14. 15610345 10.1111/j.1365-313X.2004.02272.x

[CIT0004] Chapman KD Ohlrogge JB 2012 Compartmentation of triacylglycerol accumulation in plants. The Journal of Biological Chemistry 287, 2288–2294. 22090025 10.1074/jbc.R111.290072 PMC3268389

[CIT0005] Charng YY Liu HC Liu NY Chi WT Wang CN Chang SH Wang TT 2007 A heat-inducible transcription factor, HsfA2, is required for extension of acquired thermotolerance in *Arabidopsis* . Plant Physiology 143, 251–262. 17085506 10.1104/pp.106.091322 PMC1761974

[CIT0006] Charng YY Liu HC Liu NY Hsu FC Ko SS 2006 *Arabidopsis* Hsa32, a novel heat shock protein, is essential for acquired thermotolerance during long recovery after acclimation. Plant Physiology 140, 1297–1305. 16500991 10.1104/pp.105.074898 PMC1435801

[CIT0007] Chen J Burke JJ Velten J Xin Z 2006 FtsH11 protease plays a critical role in *Arabidopsis* thermotolerance. The Plant Journal 48, 73–84. 16972866 10.1111/j.1365-313X.2006.02855.x

[CIT0008] Clarke SM Cristescu SM Miersch O Harren FJ Wasternack C Mur LA 2009 Jasmonates act with salicylic acid to confer basal thermotolerance in *Arabidopsis thaliana* . New Phytologist 182, 175–187. 19140948 10.1111/j.1469-8137.2008.02735.x

[CIT0009] El-Hafid L Pham AT Zuily-Fodil Y da Silva JV 1989 Enzymatic breakdown of polar lipids in cotton leaves under water stress: I. Degradation of monogalactosyl-diacylglycerol. Plant Physiology and Biochemistry 27.

[CIT0010] Eymery F Rey P 1999 Immunocytolocalization of CDSP 32 and CDSP 34, two chloroplastic drought-induced stress proteins in *Solanum tuberosum* plants. Plant Physiology and Biochemistry 37, 305–312.

[CIT0011] Gasulla F Vom Dorp K Dombrink I Zahringer U Gisch N Dormann P Bartels D 2013 The role of lipid metabolism in the acquisition of desiccation tolerance in *Craterostigma plantagineum*: a comparative approach. The Plant Journal 75, 726–741. 23672245 10.1111/tpj.12241

[CIT0012] Gaude N Brehelin C Tischendorf G Kessler F Dormann P 2007 Nitrogen deficiency in *Arabidopsis* affects galactolipid composition and gene expression and results in accumulation of fatty acid phytyl esters. The Plant Journal 49, 729–739. 17270009 10.1111/j.1365-313X.2006.02992.x

[CIT0013] Goni FM Alonso A 1999 Structure and functional properties of diacylglycerols in membranes. Progress in Lipid Research 38, 1–48. 10396601 10.1016/s0163-7827(98)00021-6

[CIT0014] Hong SW Vierling E 2000 Mutants of *Arabidopsis thaliana* defective in the acquisition of tolerance to high temperature stress. Proceedings of the National Academy of Sciences of the United States of America 97, 4392–4397. 10760305 10.1073/pnas.97.8.4392 PMC18252

[CIT0015] Hruz T Laule O Szabo G Wessendorp F Bleuler S Oertle L Widmayer P Gruissem W Zimmermann P 2008 Genevestigator v3: a reference expression database for the meta-analysis of transcriptomes. Advances in Bioinformatics 2008, 420747. 19956698 10.1155/2008/420747 PMC2777001

[CIT0016] Kaplan F Kopka J Haskell DW Zhao W Schiller KC Gatzke N Sung DY Guy CL 2004 Exploring the temperature-stress metabolome of *Arabidopsis* . Plant Physiology 136, 4159–4168. 15557093 10.1104/pp.104.052142 PMC535846

[CIT0017] Kaup MT Froese CD Thompson JE 2002 A role for diacylglycerol acyltransferase during leaf senescence. Plant Physiology 129, 1616–1626. 12177474 10.1104/pp.003087 PMC166749

[CIT0018] Kotak S Port M Ganguli A Bicker F von Koskull-Doring P 2004 Characterization of C-terminal domains of *Arabidopsis* heat stress transcription factors (Hsfs) and identification of a new signature combination of plant class A Hsfs with AHA and NES motifs essential for activator function and intracellular localization. The Plant Journal 39, 98–112. 15200645 10.1111/j.1365-313X.2004.02111.x

[CIT0019] Larkindale J Hall JD Knight MR Vierling E 2005 Heat stress phenotypes of *Arabidopsis* mutants implicate multiple signaling pathways in the acquisition of thermotolerance. Plant Physiology 138, 882–897. 15923322 10.1104/pp.105.062257 PMC1150405

[CIT0020] Larkindale J Knight MR 2002 Protection against heat stress-induced oxidative damage in *Arabidopsis* involves calcium, abscisic acid, ethylene and salicylic acid. Plant Physiology 128, 682–695. 11842171 10.1104/pp.010320 PMC148929

[CIT0021] Larkindale J Vierling E 2008 Core genome responses involved in acclimation to high temperature. Plant Physiology 146, 748–761. 18055584 10.1104/pp.107.112060 PMC2245833

[CIT0022] Lippold F vom Dorp K Abraham M Holzl G Wewer V Yilmaz JL Lager I Montandon C Besagni C Kessler F Stymne S Dormann P 2012 Fatty acid phytyl ester synthesis in chloroplasts of *Arabidopsis* . The Plant Cell 24, 2001–2014. 22623494 10.1105/tpc.112.095588 PMC3442583

[CIT0023] Liu HC Charng YY 2012 Acquired thermotolerance independent of heat shock factor A1 (HsfA1), the master regulator of the heat stress response. Plant Signaling and Behavior 7, 547–550. 22516818 10.4161/psb.19803 PMC3419016

[CIT0024] Liu HC Liao HT Charng YY 2011 The role of class A1 heat shock factors (HSFA1s) in response to heat and other stresses in *Arabidopsis* . Plant Cell and Environment 34, 738–751. 10.1111/j.1365-3040.2011.02278.x 21241330

[CIT0025] Martin BA Wilson RF 1984 Subcellular localization of TAG synthesis in spinach l. Lipids 19, 117–121. 10.1007/BF02534501 27520324

[CIT0026] Moellering ER Muthan B Benning C 2010 Freezing tolerance in plants requires lipid remodeling at the outer chloroplast membrane. Science 330, 226–228. 20798281 10.1126/science.1191803

[CIT0027] Murphy RC James PF McAnoy AM Krank J Duchoslav E Barkley RM 2007 Detection of the abundance of diacylglycerol and triacylglycerol molecular species in cells using neutral loss mass spectrometry. Analytical Biochemistry 366, 59–70. 17442253 10.1016/j.ab.2007.03.012 PMC2034497

[CIT0028] Nishizawa A Yabuta Y Yoshida E Maruta T Yoshimura K Shigeoka S 2006 *Arabidopsis* heat shock transcription factor A2 as a key regulator in response to several types of environmental stress. The Plant Journal 48, 535–547. 17059409 10.1111/j.1365-313X.2006.02889.x

[CIT0029] Nishizawa A Yabuta Y Shigeoka S 2008 Galactinol and raffinose constitute a novel function to protect plants from oxidative damage. Plant Physiology 147, 1251–1263. 18502973 10.1104/pp.108.122465 PMC2442551

[CIT0030] Panikulangara TJ Eggers-Schumacher G Wunderlich M Stransky H Schoffl F 2004 Galactinol synthase1. A novel heat shock factor target gene responsible for heat-induced synthesis of raffinose family oligosaccharides in *Arabidopsis* . Plant Physiology 136, 3148–3158. 15466240 10.1104/pp.104.042606 PMC523375

[CIT0031] Sakaki T Kondo N Yamada M 1990 *a* Free fatty acids regulate two galactosyltransferases in chloroplast envelope membranes isolated from spinach leaves. Plant Physiology 94, 781–787. 16667779 10.1104/pp.94.2.781 PMC1077299

[CIT0032] Sakaki T Kondo N Yamada M 1990 *b* Pathway for the synthesis of triacylglycerols from monogalactosyldiacylglycerols in ozone-fumigated spinach leaves. Plant Physiology 94, 773–780. 16667778 10.1104/pp.94.2.773 PMC1077298

[CIT0033] Sakaki T Saito K Kawaguchi A Kondo N Yamada M 1990c Conversion of monogalactosyldiacylglycerols to triacylglycerols in ozone-fumigated spinach leaves. Plant Physiology 94, 766–772. 16667777 10.1104/pp.94.2.766 PMC1077297

[CIT0034] Schneider T Keller F 2009 Raffinose in chloroplasts is synthesized in the cytosol and transported across the chloroplast envelope. Plant and Cell Physiology 50, 2174–2182. 19880397 10.1093/pcp/pcp151

[CIT0035] Schramm F Ganguli A Kiehlmann E Englich G Walch D von Koskull-Doring P 2006 The heat stress transcription factor HsfA2 serves as a regulatory amplifier of a subset of genes in the heat stress response in *Arabidopsis* . Plant Molecular Biology 60, 759–772. 16649111 10.1007/s11103-005-5750-x

[CIT0036] Steinmuller D Tevini M 1985 Composition and function of plastoglobuli: I. Isolation and purification from chloroplasts and chromoplasts. Planta 163, 201–207. 24249339 10.1007/BF00393507

[CIT0037] Taji T Ohsumi C Iuchi S Seki M Kasuga M Kobayashi M Yamaguchi-Shinozaki K Shinozaki K 2002 Important roles of drought- and cold-inducible genes for galactinol synthase in stress tolerance in *Arabidopsis thaliana* . The Plant Journal 29, 417–426. 11846875 10.1046/j.0960-7412.2001.01227.x

[CIT0038] Watanabe M Balazadeh S Tohge T Erban A Giavalisco P Kopka J Mueller-Roeber B Fernie AR Hoefgen R 2013 Comprehensive dissection of spatiotemporal metabolic shifts in primary, secondary, and lipid metabolism during developmental senescence in *Arabidopsis* . Plant Physiology 162, 1290–1310. 23696093 10.1104/pp.113.217380 PMC3707545

[CIT0039] Welti R Li W Li M Sang Y Biesiada H Zhou HE Rajashekar CB Williams TD Wang X 2002 Profiling membrane lipids in plant stress responses. Role of phospholipase D alpha in freezing-induced lipid changes in *Arabidopsis* . The Journal of Biological Chemistry 277, 31994–32002. 12077151 10.1074/jbc.M205375200

[CIT0040] Yeh CH Kaplinsky NJ Hu C Charng YY 2012 Some like it hot, some like it warm: phenotyping to explore thermotolerance diversity. Plant Science 195, 10–23. 22920995 10.1016/j.plantsci.2012.06.004 PMC3430125

[CIT0041] Yoshida T Ohama N Nakajima J Kidokoro S Mizoi J Nakashima K Maruyama K Kim JM Seki M Todaka D Osakabe Y Sakuma Y Schoffl F Shinozaki K Yamaguchi-Shinozaki K 2011 *Arabidopsis* HsfA1 transcription factors function as the main positive regulators in heat shock-responsive gene expression. Molecular Genetics and Genomics 286, 321–332. 21931939 10.1007/s00438-011-0647-7

[CIT0042] Zhang R Wise RR Struck KR Sharkey TD 2010 Moderate heat stress of *Arabidopsis thaliana* leaves causes chloroplast swelling and plastoglobule formation. Photosynthesis Research 105, 123–134. 20563644 10.1007/s11120-010-9572-6

[CIT0043] Zoeller M Stingl N Krischke M Fekete A Waller F Berger S Mueller MJ 2012 Lipid profiling of the *Arabidopsis* hypersensitive response reveals specific lipid peroxidation and fragmentation processes: biogenesis of pimelic and azelaic acid. Plant Physiology 160, 365–378. 22822212 10.1104/pp.112.202846 PMC3440211

