# Supplementary material for: Accumulation of extra-chloroplastic triacylglycerols in Arabidopsis seedlings during heat acclimation
Source: J Exp Bot. 2015 May 14;66(15):4517–26. doi: 10.1093/jxb/erv226 (PMC4507766; doi:10.1093/jxb/erv226)
Supplement: Supplementary Data [file supp_66_15_4517__index.html]

Accumulation of extra-chloroplastic triacylglycerols in Arabidopsis seedlings during heat acclimation — Accumulation of extra-chloroplastic triacylglycerols in Arabidopsis seedlings during heat acclimation — Supplementary Data 

# Accumulation of extra-chloroplastic triacylglycerols in *Arabidopsis* seedlings during heat acclimation

## Supplementary Data

Data files

**Files in this Data Supplement:**

- Supplementary Data - Supplementary Data
- Supplementary Data - Supplementary Data
