# Supplementary material for: Accumulation of extra-chloroplastic triacylglycerols in Arabidopsis seedlings during heat acclimation
Source: J Exp Bot. 2015 May 14;66(15):4517–26. doi: 10.1093/jxb/erv226 (PMC4507766; doi:10.1093/jxb/erv226)
Supplement: Supplementary Data [file supp_erv226_jexbot149674_file001.pdf]

Accumulation of extra-chloroplastic triacylglycerols in Arabidopsis seedlings during heat acclimation

Stephanie P. Mueller[1], Daniel M. Krause[1], Martin J. Mueller[1], Agnes Fekete[1]

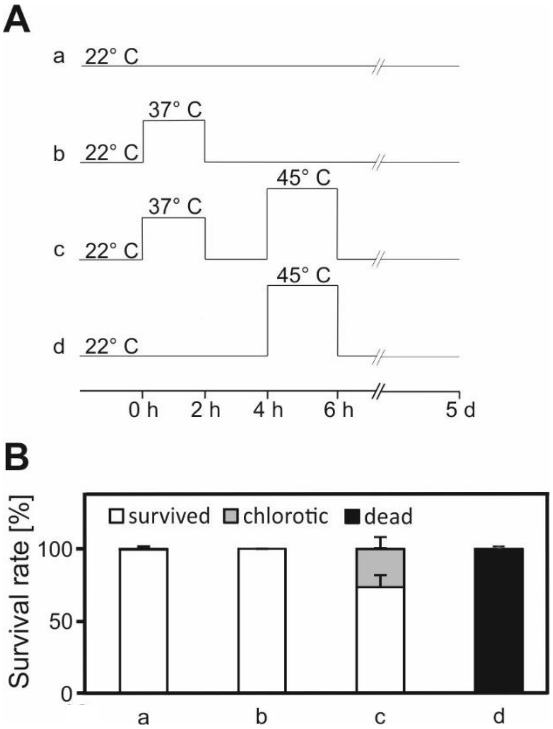

Suppl. Fig. S1. Thermotolerance assay. Schemes of heat stress regimes for the thermotolerance assay is shown in panel A. Arabidopsis seedlings (100 seed/plate) were grown at 22° C (a) and heat acclimated at 37° C for 2 h (b). Acclimated (c) and non acclimated (d) seedlings (100 seed/plate) were then shifted to 45° C for 2 h. The survival rate was measured 5 days after the heat treatment (B). Data represent means ± SD, n = 4.

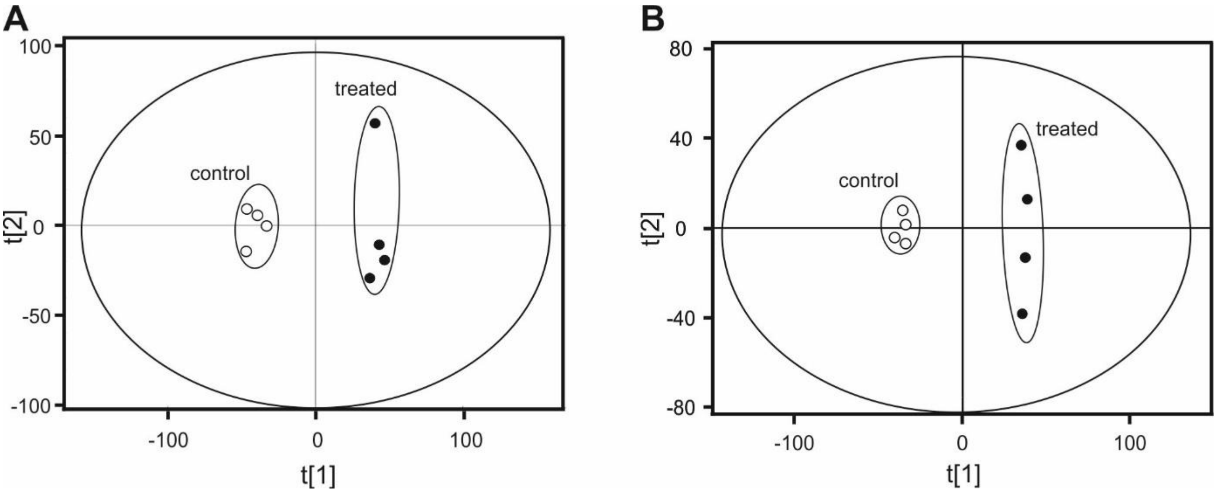

Suppl. Fig. S2. Principal components analysis (PCA) revealed a discrimination in the metabolome of heat-acclimated and control seedlings. Metabolite features of the hydrophilic (A) and hydrophobic (B) compounds were subjected to PCA. The first component in the PCA plots differentiated the metabolite features of heat-acclimated from control seedlings. n = 4.
